# Meeting of Medical Societies

**Published:** 1891-04

**Authors:** 


### Society Notes.

### Meetings of State Medical Associations.

**Washington.**—At Seattle, May 6th, 1891. We observe with pleasure, that our friend, an ex-member T. S. M. A., Dr. Wm. Caston, late of Corsicana, Texas, now of Spokane Falls, Washington, is chairman of Section on Ophthalmology and Otology.

---

**Congress of American Physicians and Surgeons.**—The meeting of the Congress of American Physicians and Surgeons will be held in Washington from 3 to 6 p. m., September 22nd, 23rd, 24th and 25th, 1891. William Pepper, chairman of Executive Committee.

---

**American Medical Association.**—Washington, D. C., May 5, 6, 7 and 8, Dr. George Dock, of Galveston, is Secretary to Section on Practice.

